# Completeness of the circle of Willis and risk of ischemic stroke in patients without cerebrovascular disease

**DOI:** 10.1007/s00234-015-1589-2

**Published:** 2015-09-10

**Authors:** Tom van Seeters, Jeroen Hendrikse, Geert Jan Biessels, Birgitta K Velthuis, Willem PTM Mali, L Jaap Kappelle, Yolanda van der Graaf, SMART Study Group

**Affiliations:** Department of Radiology, University Medical Center Utrecht, Heidelberglaan 100, HP E01.132, 3584 CX Utrecht, The Netherlands; Department of Neurology, Brain Center Rudolf Magnus, University Medical Center Utrecht, Utrecht, The Netherlands; Julius Center for Health Sciences and Primary Care, University Medical Center Utrecht, Utrecht, The Netherlands

**Keywords:** Ischemic stroke, Circle of Willis, Carotid stenosis

## Abstract

**Introduction:**

We investigated circle of Willis (CoW) completeness in relation to the risk of future ischemic stroke in patients without prior cerebrovascular disease.

**Methods:**

We included 976 patients with atherosclerotic disease, but no previous TIA/stroke, from the Second Manifestations of ARTerial disease (SMART) study. All patients underwent MR angiography of the CoW. Cox regression was used to determine whether anterior CoW completeness (anterior communicating artery or A1 segments) and posterior CoW completeness (posterior communicating arteries or P1 segments) were related to future stroke, and whether CoW completeness influenced the relation between internal carotid artery (ICA) stenosis/occlusion and future stroke.

**Results:**

Thirty patients (3.1 %) had ischemic stroke after 9.2 ± 3.0 years of follow-up. Twenty-four patients (80 %) had anterior circulation stroke. An incomplete anterior CoW was related to future anterior circulation stroke (HR 2.8 (95 % CI 1.3–6.3); *p* = 0.01), whereas a one-sided and two-sided incomplete posterior CoW were not (HR 2.2 (95 % CI 0.7–7.1; *p* = 0.19) and 1.9 (95 % CI 0.6–5.9; *p* = 0.29), respectively). In stratified analyses, patients with an incomplete anterior CoW had the highest risk of future anterior circulation stroke when they also had a one-sided (HR 7.0 (95 % CI 1.3–38.2; *p* = 0.02)) or two-sided incomplete posterior CoW (HR 5.4 (95 % CI 1.0–27.8; *p* = 0.04). CoW completeness did not change the relation between asymptomatic ICA stenosis/occlusion and future ischemic stroke (*p* = 0.68).

**Conclusions:**

An incomplete anterior CoW combined with an incomplete posterior CoW is related to future anterior circulation stroke. CoW completeness has no large effect on the relation between asymptomatic ICA stenosis/occlusion and future stroke.

## Introduction

The circle of Willis (CoW) provides several pathways for collateral blood flow to the brain [1], but CoW segments are frequently hypoplastic or absent [1, 2]. Previous research suggests a relation between CoW completeness and risk of future ischemic stroke for patients with symptomatic cerebrovascular disease [3–5]. In these patients, patent CoW collaterals are thought to protect against future ischemic stroke by providing increased collateral flow. However, this has only been investigated in patients with a severe stenosis or occlusion of the internal carotid artery (ICA).

We hypothesized that collateral flow through CoW collaterals could also be protective for stroke in high-risk patients without symptomatic cerebrovascular disease. Furthermore, asymptomatic patients with an ICA stenosis/occlusion could possibly remain asymptomatic because of patent CoW collaterals.

Therefore, we determined whether CoW completeness is related to future ischemic stroke in patients at high cardiovascular risk, but without previous TIA/stroke. Subsequently, we investigated if CoW completeness changed the relation between asymptomatic ICA stenosis/occlusion and ischemic stroke.

## Methods

### Study population

**Fig. 1:**
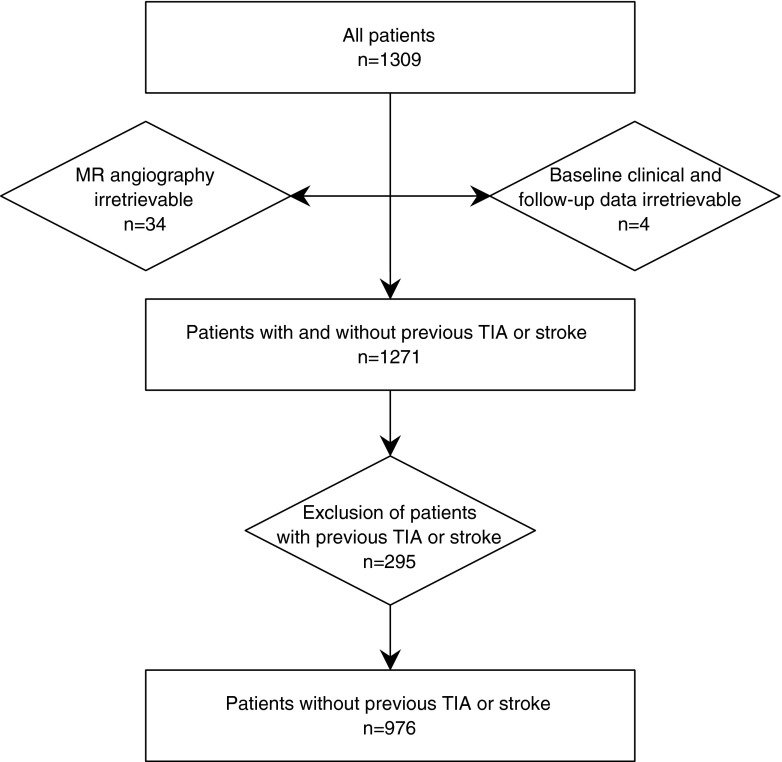
Flowchart

Patients participated in the Second Manifestations of ARTerial disease – Magnetic Resonance (SMART-MR) study, a prospective cohort study in patients with atherosclerotic disease. The study protocol has been described in detail elsewhere [6, 7]. Between May 2001 and December 2005, 1309 patients were enrolled with manifest coronary artery disease, cerebrovascular disease, peripheral arterial disease, or an abdominal aortic aneurysm, and without contraindications for MRI. All patients underwent MR angiography (MRA) of the CoW. For the present study, four patients (0.3 %) were excluded because baseline and follow-up information was not available, 34 patients (2.6 %) because the MRA was irretrievable, and 295 patients (22.5 %) because they had cerebrovascular disease in their medical history. Hence, our study population consisted of 976 patients without a previous TIA or stroke (Fig. 1). The medical ethics committee of the University Medical Center Utrecht approved the study. Written informed consent was obtained from all participants.

### CoW assessment

The CoW was assessed with time-of-flight MRA, performed at baseline. All MRI investigations were performed on a 1.5-T Philips Gyroscan (Gyroscan ACS-NT, Philips Medical Systems, Best, The Netherlands). The MRA consisted of 50 slices obtained with a 3D MRA time-of-flight technique (TR/TE 31/6.9 ms, flip angle 20, 2 signals acquired, slice thickness of 1.2 mm with an overlap of 0.6 mm, field of view 100 × 100 mm, matrix size 128 × 128). Images were reconstructed in the transversal-oblique plane with a maximum intensity projection algorithm.

The CoW was assessed by two experienced observers (JH and AFvR), who used the thin slice source MRA data in combination with maximum intensity projections. The anterior CoW was incomplete if the anterior communicating artery or A1 segment(s) was hypoplastic (<0.8 mm) or absent. The posterior CoW was incomplete if one of the posterior communicating arteries or P1 segments was hypoplastic (<0.8 mm) or absent in either hemisphere [2]. The anterior CoW was classified as either complete or incomplete. The posterior CoW was classified as complete, one-sided incomplete, or two-sided incomplete.

### Assessment of the ICA

The degree of ICA stenosis was determined at baseline with color Doppler-assisted duplex scanning of the carotid arteries. The degree of stenosis or occlusion was classified according to previously published thresholds for blood flow velocities [8]. Severe ICA stenosis or occlusion was defined as presence of either a >70 % ICA stenosis (peak systolic velocity (PSV) >270 cm/s), pre-occlusion (PSV >270 cm/s and distal PSV <40 cm/s), subtotal stenosis (PSV <50 cm/s and severe plaque), or occlusion (no flow). As patients with prior cerebrovascular disease were excluded from this study, all stenoses and occlusions of the ICA were asymptomatic.

### Outcome

The study outcomes were occurrence of ischemic stroke in general, and occurrence of ischemic stroke in the anterior circulation. For outcome assessment, patients completed a questionnaire on hospitalizations and outpatient clinic visits on a half yearly basis. When a possible event was recorded, hospital discharge letters and other correspondence and investigations relating to the event were collected [6]. Ischemic stroke was defined as having clinical features of sudden onset for ≥24 h, with increased impairment of ≥1 point on the modified Rankin Scale [9], with or without documentation of a new infarct on CT/MRI, and no other potential cause than ischemic stroke. If the infarct occurred within the anterior circulation, it was classified as an anterior circulation stroke. Follow-up duration was defined as the period between inclusion date and either date of event, or date of loss-to-follow-up. If patients with an ICA stenosis underwent carotid endarterectomy, follow-up was censored from that moment.

### Analyses

Single imputation was performed for missing information on ICA stenosis/occlusion (*n* = 36, 3.7 %). CoW data were complete. Cox proportional hazards analysis was used to determine the relation between CoW completeness and future ischemic stroke. Next, stratified analyses were performed combining completeness of the anterior and posterior CoW. Cox proportional hazards analysis was also used to assess whether CoW completeness changed the relation between asymptomatic ICA stenosis/occlusion and ischemic stroke, after adjusting for age and gender. Analyses were performed with R version 3.0.2.

## Results

During 9003 follow-up years (mean patient follow-up 9.2 ± 3.0 years), 30 patients (3.1 %) suffered from ischemic stroke (3.3/1000 person-years), of whom 24 patients (80 %) had an anterior circulation stroke. Additional patient characteristics are presented in Table 1. The cardiovascular risk factor profile was similar for patients with incomplete and complete CoW configurations.

**Table 1:** Patient characteristics (*n* = 976)

Age (years)	58.2 (9.9)
Male gender	782 (80.1)
Current smoking	336 (34.7)
Past smoking	452 (46.7)
Glucose (mmol/L)	5.8 (5.3–6.5)
Systolic blood pressure (mmHg)	140 (20.8)
Diastolic blood pressure (mmHg)	82 (10.9)
Medical history	
Coronary artery disease	687 (70.4)
Peripheral arterial disease	246 (25.2)
Abdominal aortic aneurysm	103 (10.6)
Hypertension	466 (48.3)
Hyperlipidemia	767 (79.5)
Diabetes	145 (15.8)
CoW completeness	
Incomplete anterior CoW	223 (22.8)
One-sided incomplete posterior CoW	291 (29.8)
Two-sided incomplete posterior CoW	398 (40.8)
>70 % stenosis/occlusion of internal carotid artery	43 (4.6)
Study outcomes	
Ischemic stroke	30 (3.1)
Anterior circulation stroke	24 (2.5)
Follow-up (years)	9.2 (3.0)

Data are displayed as mean (standard deviation), median (interquartile range), or *n* (%)

*CoW* circle of Willis

Ischemic stroke occurred in 11/223 patients (4.9 %) with an incomplete anterior CoW and in 19/753 patients (2.5 %) with a complete anterior CoW. The corresponding hazard ratio (HR) was 1.9 (95 % CI 0.9–4.1; *p* = 0.08; Table 2). Regarding posterior CoW completeness, 13/398 patients (3.3 %) with a two-sided incomplete posterior CoW, 10/291 patients (3.4 %) with a one-sided incomplete posterior CoW, and 7/287 patients (2.4 %) with a complete posterior CoW suffered from ischemic stroke. The corresponding HR was 1.3 (95 % CI 0.5–3.2; *p* = 0.62) for patients with a two-sided incomplete posterior CoW and 1.4 (95 % CI 0.5–3.6; *p* = 0.51) for patients with a one-sided incomplete posterior CoW.

**Table 2:** Hazard ratios (95 % confidence interval) for the relation between CoW completeness and future ischemic stroke, after a mean follow-up of 9.2 ± 3.0 years (*n* = 976)

	Ischemic stroke (*n* = 30)	Anterior circulation stroke (*n* = 24)
Completeness of individual CoW collateral pathways		
Anterior CoW completeness		
Complete anterior CoW (*n* = 753)	1.0 (reference)	1.0 (reference)
Incomplete anterior CoW (*n* = 223)	1.9 (0.9–4.1)	2.8 (1.3–6.3)*
Posterior CoW completeness		
Complete posterior CoW (*n* = 287)	1.0 (reference)	1.0 (reference)
One-sided incomplete posterior CoW (*n* = 291)	1.4 (0.5–3.6)	2.2 (0.7–7.1)
Two-sided incomplete posterior CoW (*n* = 398)	1.3 (0.5–3.2)	1.9 (0.6–5.9)
Completeness of combined anterior and posterior CoW collateral pathways		
Complete anterior and posterior CoW (*n* = 233)	1.0 (reference)	1.0 (reference)
Complete anterior CoW, one-sided incomplete posterior CoW (*n* = 225)	1.2 (0.4–3.9)	2.5 (0.5–12.9)
Complete anterior CoW, two-sided incomplete posterior CoW (*n* = 295)	1.2 (0.4–3.6)	2.2 (0.4–10.9)
Incomplete anterior CoW, complete posterior CoW (*n* = 54)	1.6 (0.3–8.3)	4.0 (0.6–28.6)
Incomplete anterior CoW, one-sided incomplete posterior CoW (*n* = 66)	2.8 (0.7–10.3)	7.0 (1.3–38.2)*
Incomplete anterior CoW, two-sided incomplete posterior CoW (*n* = 103)	2.1 (0.6–7.4)	5.4 (1.0–27.8)*

*CoW* circle of Willis

*p < 0.05

Presence of an incomplete anterior CoW was related to occurrence of anterior circulation stroke (HR 2.8 (95 % CI 1.3–6.3); *p* = 0.01), whereas presence of either a one-sided or two-sided incomplete posterior CoW was not related to anterior circulation stroke (HR 2.2 (95 % CI 0.7–7.1; *p* = 0.19) and 1.9 (95 % CI 0.6–5.9; *p* = 0.29), respectively). However, additional stratified analyses revealed that patients with an incomplete anterior CoW had the highest risk of anterior circulation stroke if the posterior CoW was incomplete as well (Table 2). This is shown by the HR of 7.0 (95 % CI 1.3–38.2; *p* = 0.02) for patients with an incomplete anterior and one-sided incomplete posterior CoW, and the HR of 5.4 (95 % CI 1.0–27.8; *p* = 0.04) for patients with an incomplete anterior and two-sided incomplete posterior CoW.

Patients with an asymptomatic ICA stenosis/occlusion had an incomplete anterior CoW (*n* = 10, 23 % (95 % CI 11–36 %)) equally often as patients without an ICA stenosis/occlusion (*n* = 206, 23 % (95 % CI 20–26 %)), whereas patients with an asymptomatic ICA stenosis/occlusion less frequently had an incomplete posterior CoW (*n* = 21, 49 % (95 % CI 34–64)) than patients without an ICA stenosis/occlusion (*n* = 646, 72 % (95 % CI 69–75); *p* = 0.001). The HR for the relation between asymptomatic ICA stenosis/occlusion and ischemic stroke was 3.6 (95 % CI 1.2–10.5) after adjustment for age and gender, and did not change after additional adjustment for CoW completeness (HR 4.1 (95 % CI 1.4–12.4); *p* = 0.68; Table 3).

**Table 3:** Relation between asymptomatic ICA stenosis/occlusion and stroke, before and after adjustment for CoW completeness

Outcome	Number of events/*n*	Crude HR (95 % CI)	HR adjusted for age and gender (95 % CI)	HR adjusted for age, gender, and CoW completeness (95 % CI)
Ischemic stroke	30/976	4.7 (1.6–13.4)	3.6 (1.2–10.5)	4.1 (1.4–12.4)
Anterior circulation stroke	24/976	6.0 (2.1–17.7)	4.7 (1.6–14.0)	6.1 (2.0–18.8)

*ICA* internal carotid artery, *CoW* circle of Willis, *HR* hazard ratio, *CI* confidence interval

## Discussion

In patients without a history of cerebrovascular disease, we showed that completeness of the anterior CoW was related to the occurrence of anterior circulation stroke. In stratified analyses combining completeness of the anterior and posterior CoW, anterior circulation stroke risk was highest if both the anterior and posterior CoW were incomplete. CoW completeness did not strongly affect the increased risk of future stroke in patients with asymptomatic carotid disease.

The results of our study should be interpreted with some caution. Thirty patients had an ischemic stroke during follow-up with a corresponding incidence rate of 3.3/1000 person-years, which is consistent with previous studies [10]. However, the number of patients with stroke was lower in the six strata that were used in the stratified analyses combining anterior and posterior CoW completeness. Despite 9003 follow-up years, our study may therefore suffer from lack of power to demonstrate weaker associations, for example between posterior CoW completeness and future anterior circulation stroke in patients with a complete anterior CoW.

The relation between CoW completeness and ischemic stroke has been investigated previously in patients with ICA stenosis [4, 11] or occlusion [3, 5, 11, 12], but only once in patients without prior cerebrovascular disease [13]. In this previous cross-sectional case–control study, the proportion of patients with an incomplete posterior CoW (43 %) was lower than in our prospective cohort study (71 %). This may be partially explained by the higher proportion of patients with ICA stenosis/occlusion in the previous study (26 %) compared to our study (5 %), as we showed that these patients more often have a complete posterior CoW. This in turn may be explained by recruitment (increased flow) through a hypoplastic posterior CoW to compensate for decreased blood supply from the ICA [12]. Nonetheless, in this previous study, completeness of the anterior CoW was also related to ischemic stroke, but analyses combining completeness of the anterior and posterior CoW were not performed.

The CoW has long been viewed primarily as a collateral conduit. Recently, alternative views have been put forward [14]. Arguments are that, from an evolutionary point of view, it is unlikely that the CoW solely developed to compensate for abnormal blood supply in case of vessel occlusions, since pathologic conditions that mainly occur in the elderly cannot steer evolution. From a physiological point of view, some believe that the communicating arteries are too small for effective collateral blood supply. It has been suggested that the CoW may be seen as passive energy dissipating system that equalizes the pressure wave in the brain [14]. This alternative view should be taken into account when interpreting studies about the CoW.

Strengths of our study are the large sample size, prospectively collected data, and long follow-up duration, but our study also has some limitations. A more accurate assessment of the CoW could be achieved by using conventional angiography instead of MRA. CoW segments are often small, and MRA may therefore have underestimated the proportion of patients with a complete CoW. Hence, patients with a complete CoW according to conventional angiography could have been classified as having an incomplete CoW on MRA. However, we considered it unfeasible to perform conventional angiography in our cohort of asymptomatic patients. Another limitation is that information on stroke etiology other than ICA disease (e.g., cardiac embolism) was not assessed.

In conclusion, our study shows that an incomplete anterior CoW combined with an incomplete posterior CoW is related to the occurrence of future anterior circulation stroke. CoW completeness has no large effect on the relation between asymptomatic ICA stenosis/occlusion and ischemic stroke.
